# Investigating pupillometry to detect preoperative anxiety: a pilot study

**DOI:** 10.3389/fpsyg.2023.1296387

**Published:** 2024-01-03

**Authors:** Jason Richez, Pierre-Raphaël Rothschild, Christophe Baillard, Gilles Guerrier

**Affiliations:** ^1^Department of Anaesthesia and Intensive Care, Hopital Cochin, Assistance Publique-Hopitaux de Paris, Paris, France; ^2^Department of Ophtalmology, Hôpital Cochin, Assistance Publique-Hôpitaux de Paris, Paris, France

**Keywords:** cataract surgery, coefficient of variation, objective assessment, pupillometry, pre-operative anxiety

## Abstract

Guidelines from the European Society of Anesthesia (ESA) insist on the importance of preoperative anxiety management. However, its assessment currently relies on questionnaires that are long to submit and sometimes difficult to interpret. Exploring the balance between sympathetic and parasympathetic neural systems through the use of pupillometry is a promising path to identify anxiety and thus provides an objective and reproducible assessment tool. A single-center prospective observational study was conducted in a population of ambulatory ophthalmological surgery patients. Preoperative anxiety was assessed using the Surgical Fear Questionnaire (SFQ). Measurements were taken using an Algiscan^®^ (IDMed) type pupillometer before, during, and after insertion of the peripheral IV catheter. A statistical correlation test was carried out between the different evaluations of anxiety and the coefficient of variation of the pupillary diameter (VCPD). A total of 71 patients were included in the study between July 2020 and February 2021, with a median SFQ score of 23 [IQR 11-34]. No significant statistical correlation was found between the baseline pupillary diameter, or VCPD, and preoperative anxiety levels. Similarly, the pupillometric variables did not differ significantly when adjusting for the level of anxiety during and after painful stimulation due to canulation. More studies are necessary to explore the potential correlation between preoperative anxiety and pupillometry.

## Background

Preoperative anxiety is associated with a number of complications, including higher acute postoperative pain and increased pain at home incidence (Kain et al., 2000). Its prevalence in adults is estimated at 60%–80% in studies (Mackenzie, 1989; Shevde and Panagopoulos, 1991). While the European Society of Anesthesia (ESA) guidelines insist on the importance of managing preoperative anxiety (de Hert et al., 2018), this aspect is often neglected for various reasons, one of which is that physicians lack objective assessment for anxiety. In particular, when using a simple numerical scale (ENS), the correlation between the patient's self-assessment and the one made by the caregiver is often poor (Laufenberg-Feldmann and Kappis, 2013). A more precise assessment of the level of anxiety is based on self-assessment questionnaires, which are time-consuming for nursing staff and difficult to interpret among patients presenting communication difficulties (such as language barriers or neurocognitive disorders) or who are in denial of their anxiety. To overcome such difficulties, some objective measures of anxiety could be useful in medical settings, just as objective measures of pain are now being determined.

Changes in the state of neurovegetative activation are described during anxiety, including changes in heart rate variability (Chalmers et al., 2014), but also pupillary diameter variations occurring in many circumstances, including sensory, emotional, or cognitive stimuli (Mathôt, 2018). Different pupillometric parameters have been used and validated for assessing pain, including the variation coefficient of pupillary diameter (VCPD), which has shown excellent performance in monitoring nociception in patients both awake (Charier et al., 2017) and after general anesthesia (Charier et al., 2019). The VCPD is defined as the ratio of the median deviation to the median. It describes pupillary diameter fluctuations around the median value for the 10-s recording. Higher values of VCPD reflect higher sympathetic activity, meaning higher anxiety or pain when transposed to physiological contexts. Given the prolific literature describing other pupillometric parameters (including pupil diameter and pupil dilation) linked to emotional stimuli (in particular anxiety), the use of this device in conscious patients in the preoperative period could allow reliable and objective screening of anxiety while monitoring its variations over time.

## Methods

A single-center prospective observational study was conducted in a population of ambulatory ophthalmological surgery patients. Preoperative anxiety was assessed using different scales: the Numeric Rating Scale (NRS), the Surgical Fear Questionnaire short term (SFQ-s), and the Spielberger State and Trait Anxiety Index Y-state (STAI Y-state). Pupillometric measurements including baseline pupillary diameter (PD), PD variation during canulation, and VCPD were taken using an Algiscan® (IDMed) type pupillometer before, during, and after insertion of the peripheral IV catheter, each of them lasting 10–20 s. Measures were performed before the administration of premedication for anxiety and pupil dilation in the non-operated eye. Pupillometric measurements were taken on patients before the administration of an anxiolytic premedication, specifically oral midazolam. These patients were comfortably seated in a waiting room chair before being transferred to the operating room. A statistical correlation test was carried out between the different evaluations of anxiety and pupillometric parameters (PD, PLR, and VCPD). Patients were divided into a low-anxiety group and a high-anxiety group. The characteristics of these two groups were compared, including pupillometric variables using Student's t-test after normality was checked through a Shapiro–Wilk test or Mann–Whitney *U*-test for non-parametric variables. The number of subjects to be included in our study was based on personal data showing that the VCPD of patients in the waiting room was on an average of 1.9 (SD 1.5) for those with an SFQ of <30 and 3.2 (1.9) for those with an SFQ of ≥30. Considering an *α*-risk of 0.05 and a *ß*-power of the statistical test of 0.90, 72 patients were needed to show a significant difference between the two groups, based on an estimated VCPD standard deviation of 1.7.

## Results

**Figure 1:**
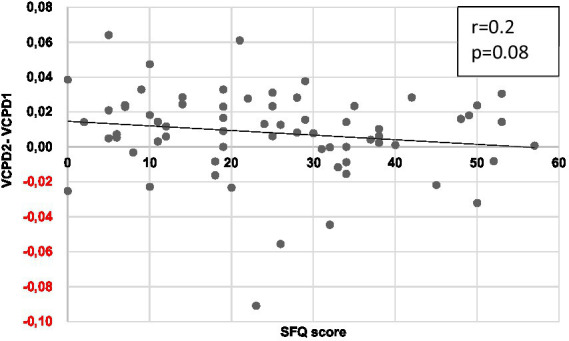
Graph showing the correlation between SFQ and VCPD variation before (VCPD1) and during canulation (VCPD2). *r*, Pearson’s coefficient of correlation; VCPD, variation coefficient of pupillary diameter. *r* = 0.2. *p* = 0.08.

With approval from the Ethics Committee (IDRCB 2019-A01743-54), 71 patients having elective cataract surgery under local anesthesia were included in the study between July 2020 and February 2021, with a median SFQ score of 23. The median age in our population was 66 [IQR 56–74]. The VCPD before, during, and after insertion of the IV catheter was 2.3%, 4.2%, and 2.7%, respectively. No significant correlation was found between those three VCPD measures and preoperative anxiety assessed by SFQ (*r* = 0.2; *p* = 0.08) (Figure 1) or STAI Y-state scores. In particular, VCPD before nociceptive stimulation (VCPD1) in anxious and non-anxious people did not differ for high, moderate, and low cutoff anxiety used (Table 1). Similarly, VCPD after nociceptive stimulation (VCPD3) of anxious and non-anxious people did not differ for all anxiety cutoffs used (Table 1).

**Table 1:** Linear correlation between the surgical fear questionnaire (SFQ) and pupillometric parameters according to anxiety level cutoffs.

	VCPD1 (SD) (%)	VCPD2 (SD) (%)	VCPD3 (SD) (%)	Pupil dilation during cannulation (%)	PD1 (mm)	PD2 (mm)
*n* = 71	2.3 (1.8)	4.2 (2.8)	2.7 (3.1)	20.7 (30.4)	2.59 (0.85)	2.46 (08)
SFQ > 30 (*n* = 24)	2.1 (0.9)	4.05 (3.37)	2.21 (1.04)	20.73 (23.67)	2.61 (0.53)	2.54 (0.54)
SFQ ≤ 30 (*n* = 47)	2.3 (1.19)	4.25 (2.43)	2.94 (3.52)	20.75 (33.55)	2.58 (0.98)	2.43 (0.89)
*p*-value	1	0.28	0.52	0.64	0.1	0.25
	VCPD1 (SD) (%)	VCPD2 (SD) (%)	VCPD3 (SD) (%)	Pupil dilation during cannulation (%)	PD1 (mm)	PD2 (mm)
*n* = 71	2,3 (1,8)	4,2 (2,8)	2.7 (3.1)	20.7 (30.4)	2.59 (0.85)	2,46 (08)
SFQ > 30 (*n* = 24)	2,1 (0,9)	4,05 (3,37)	2.21 (1.04)	20.73 (23.67)	2.61 (0.53)	2.54 (0.54)
SFQ ≤ 30 (*n* = 47)	2,3 (1,19)	4,25 (2,43)	2.94 (3.52)	20.75 (33.55)	2.58 (0.98)	2.43 (0.89)
*P*-value	1	0,28	0.52	0.64	0.1	0.25

Data are presented as mean (standard deviation); PD, pupillary diameter before (PD1) and after canulation (PD2); VCPD, variation coefficient of pupillary diameter variation before (VCPD1), during (VCPD2), and after canulation (VCPD3).

## Discussion

To date, the VCPD has been validated as a measure of pain in two conditions: during obstetrical labor (Charier et al., 2017) and in the recovery room postoperatively (Charier et al., 2019). It increases during a nociceptive stimulus with constant analgesia (Charier et al., 2017) and decreases in the absence of a nociceptive stimulus or during the administration of analgesics (Charier et al., 2019). The VCPD does not take into account the variation of the pupillary diameter (Charier et al., 2017). VCPD1 was recorded over 10 s before venipuncture, and VCPD3 was recorded over 10 s after venipuncture, but the venipuncture itself seems to us to be too short to allow the acquisition of VCPD2, probably explaining the result very close to that of VCPD1. In our study, the VCPD is almost multiplied by two when the intravenous catheter is inserted (from 2.3% to 4.2%), indicating the activation of the sympathetic system related to the painful gesture, which confirms the interest of the VCPD in measuring pain in conscious patients.

Regarding baseline pupillary diameter, there was no significant difference in this pupillometric parameter between low and high anxiety groups using both the SFQ and STAI Y-state scales at the various defined cutoffs. Similarly, there was no significant difference in pupillary dilation both before and after painful stimulation between low and high anxiety groups at the various defined cutoffs.

Since our study population presented a median SFQ score of 23 [IQR 11–34], it can be hypothesized that this level was too low overall to detect a significant difference in pupillometric parameters. The quality of the measurements in our study can also be questioned. We found indeed an average VCPD of approximately 2%, while it was 4% at rest and 9% on average with painful stimulation in the study by Charier (Charier et al., 2017). The low intensity of painful stimuli may explain the lower values observed in our study compared to those by Charier et al. as well as the lack of significance in our correlation statistics. The mean age of our study population should also be mentioned since pupil diameter variations are known to decrease with age. Finally, reporting biases are always a limitation to be mentioned when dealing with validated questionnaires on anxiety. Interestingly, a study by Ginton et al. (2022) found similar negative results of baseline pupillary diameter and dilation regarding patients with post-traumatic stress disorder (PTSD) self-reporting their emotional regulation.

To the best of our knowledge, our study is the first to assess pupillometry in screening for preoperative anxiety. Despite this negative result and considering the substantial literature describing pupillary dilation reflexes linked to cognitive and emotional stimuli, it seems that further investigations should be performed in younger patients waiting for more invasive surgery to determine whether or not pupillometry may be routinely used to measure preoperative anxiety levels.

## Data availability statement

The raw data supporting the conclusions of this article will be made available by the authors, without undue reservation.

## Ethics statement

The studies involving humans were approved by the Ethics Committee (IDRCB 2019-A01743-54). The studies were conducted in accordance with the local legislation and institutional requirements. The participants provided their written informed consent to participate in this study.

## Author contributions

JR: Conceptualization, Formal analysis, Investigation, Methodology, Project administration, Supervision, Validation, Writing – original draft, Writing – review & editing. P-RR: Investigation, Writing – original draft. CB: Methodology, Project administration, Resources, Writing – review & editing. GG: Formal analysis, Investigation, Software, Writing – review & editing.
